# Factors Influencing Child Welfare Clinic Attendance in a Periurban Community: A Descriptive Cross-Sectional Study

**DOI:** 10.1155/tswj/9914853

**Published:** 2025-02-13

**Authors:** Judith A. Torgbor-Anaman, Beatrice B. Johnson, Vivian Tackie, Kennedy Diema Konlan

**Affiliations:** ^1^Department of Public Health Nursing, School of Nursing and Midwifery, University of Health and Allied Sciences, Volta Region, Ho PMB31, Ghana; ^2^Department of Nursing, School of Nursing and Midwifery, University of Health and Allied Sciences, Volta Region, Ho PMB31, Ghana

**Keywords:** caregivers, child welfare clinics, development, growth, immunization

## Abstract

**Introduction:** Child welfare clinics (CWCs) provide a platform for health practitioners to communicate with caregivers and provide growth monitoring, childhood immunization, health education, and other health promotion services. This study described factors influencing caregivers' attendance at CWC in the Godokpe Community in Ho.

**Methodology:** This is a cross-sectional study that used questionnaires for data collection among 403 caregivers having children under 5 years. The respondents were selected using convenience sampling techniques. Data were analyzed using SPSS version 25 to generate descriptive statistics and to test associations between independent variables and CWC attendance. A *p* value ≤ 0.05 was statistically significant.

**Results:** The findings indicated that 80.4% of the caregivers had a high level of knowledge about CWC. The factors that influenced continued CWC attendance were encouragement by nurses (94.3%), the nurses being empathetic (93.8%), nurses showing a positive attitude toward caregivers (91.8%), the perception that attending CWC is an ideal childcare process (91.6%), having less waiting time (90.8%), having knowledge on child care practices (90.6%), having an appropriate timing for CWC services (90.1%), perception that there is the provision of adequate care for sick children at CWC (89.8%), perception that CWC is a needful care practice for children (82.4%), and acknowledging CWC as a requirement stated in the child welfare card (82.1%). Also, caregivers (79.0%) attended CWC sessions regularly. The adjusted odds ratio showed that caregivers without formal education (AOR = 0.10, 95% CI: 0.02–0.37, *p* value = 0.001), having primary education (AOR = 0.13, 95% CI: 0.04–0.37, *p* value < 0.001), and having secondary education (AOR = 0.34, 95% CI: 0.12–0.91, *p* value = 0.036) predicted CWC attendance compared to those with tertiary education. Experience at CWC (AOR = 2.52, 95% CI: 1.20–5.81, *p* value = 0.021) and having children between 0 and 11 months (AOR = 3.16, 95% CI: 1.50–6.89, *p* value = 0.003) predicted CWC attendance.

**Conclusion:** We identified various factors (sociodemographic and knowledge/perception) influencing CWC attendance. Healthcare providers must institute interventions targeting parents having lower education status and having children older than 11 months for continued CWC attendance even after the completion of routine immunizations. This may include continued home visits to provide CWC care to children less than 5 years old.

## 1. Introduction

Many countries around the world have prioritized the promotion of children's health in their developmental plans [1, 2]. Ghana, as a developing country, is also making deliberate efforts to promote children's health through the active implementation of programs that may contribute to the Sustainable Development Goal (SDG) 3.2 [3, 4]. This will help mitigate preventable deaths of children under five years of age by 2030 [5]. As of 2010, Ghana had a population of over 3.4 million below the age of 5 years [6] with under-five mortality in 2016 being 41/1000 live births [7] and 5.2 million in 2019 [2, 8]. Also, preventable and treatable causes of under-five mortality were attributable to 1.5 million deaths in children aged a month to a year, 1.3 million in 1 to 4 years, and 2.4 million among newborns under 28 days [2]. Most of the leading causes of death in children under 5 years are preventable and/or treatable and include preterm, birth complications, birth asphyxia/trauma, pneumonia, congenital anomalies, diarrhea, low birth weight, early and late maternal age, maternal multiple pregnancies, and maternal malaria [2, 7, 9].

Measures have been implemented to curb the mortality among children under 5 years including the introduction and use of child welfare clinics (CWCs), especially in resource-limited settings. CWC is an essential component of Ghana's primary healthcare system commissioned to ensure effective growth monitoring, immunizations, deworming, screening for malnutrition, case management, and educating caregivers on health promotion practices that support child development [1, 10, 11]. In addition, CWC provides an important platform for health practitioners to communicate with caregivers and establish consensus on better and practical approaches to promote optimal child development [1, 10, 11]. It is recommended that mothers complete all 60 months of required CWC attendance for each child. Although child immunization is completed at the age of 24 months in Ghana, additional vital services such as growth monitoring, deworming, screening for malnutrition, case management, and referral are provided until the child reaches the age of 60 months [5, 12]. Similarly, during this period, emerging health issues are addressed so that they do not progress into more serious concerns [13, 14]. However, in most underserved communities in Ghana, CWC attendance often drops after 24 months when all routine immunization schedules have been completed [15].

Despite the multiple benefits of CWC, there was reported low use, especially among people in rural and periurban areas, and mostly after the mandatory immunizations at 24 months [15–17]. In rural Ghana, the mean annual attendance at CWC was six visits per year [15, 18]. Over a decade ago, the Ghana Health Service reported a steady drop in CWC attendance, which was related to caregivers' knowledge gap and the lack of tracing systems for defaulters [19]. The gap in attendance is even higher in rural communities where continued monitoring is expected to be imperative because of poor health knowledge, limited healthcare access, inadequate use, and higher influence of inimical cultural practices. The factors responsible for these drastic changes in CWC attendance have not been identified in recent times in most rural and periurban settings in Ghana. To curtail the inadequate CWC attendance after 24 months in rural and periurban communities, practitioners and policymakers must institute practical measures to promote and address the barriers. This will warrant that research focuses on identifying and outlining the sociodemographic and behavioral factors that hinder low attendance, especially in children less than 5 years old. Therefore, this study aimed to describe the factors influencing CWC attendance in a periurban community in the Ho municipality.

## 2. Materials and Methods

### 2.1. Research Design

This research used a cross-sectional design that allowed the researchers to compare multiple variables through data collection at a single point in time without follow-up.

### 2.2. Study Setting

This study was conducted in Godokpe, a suburb of Ho in the Volta Region of Ghana. Volta Region is one of the Ghana's 16 administrative regions, with Ho designated as its capital. The region is divided into 25 administrative districts, multiethnic and multilingual. The languages spoken mostly include Ewe, Guan, Hausa, and Akan. The Godokpe community is located in the eastern part of Ho township, and it is home to the Ho Polyclinic, which serves as the main center for CWC service provision. The CWC at the Ho Polyclinic offers both static and outreach services to the surrounding communities. These outreach services are provided to Titrinu, New Town, Dornorkordzi, Dave, Lepers Village, Dokpo Kope, and Desiadenyo.

### 2.3. Population, Sampling, and Sample Size Estimation

The target population was all caregivers having children less than or equal to five years (60 months) in the Godokpe community. A convenience sampling method was used to select the caregivers in the community. The convenience sampling method is a nonprobability sampling method where units are selected for inclusion based on their immediate availability. Caregivers who were readily available and willing to participate in this study were selected through a house-to-house visit. The children's ages were confirmed using the CWC attendance book, birth certificate, school attendance record, and/or collaborative confirmation by at least two adults within the household. Recruited research assistants administered the questionnaires for the data collection.

The sample size for the study was calculated using the Cochran formula for calculating sample sizes for infinite populations at a 95% confidence interval, *n* = *Z*^2^*pq*/*d*^2^, where *n* is the required sample size; *Z* = *Z* statistic for a level of confidence at 95% with a standard value of 1.96, *Z*^2^ = 1.96^2^ = 3.8416; *p*=(0.5) estimated proportion of caregivers available for data collection, *q* = 1 − *p* = 0.5; *d* = margin of error = 5% = 0.05; and *d*^2^ = 0.05^2^ = 0.0025. Substituting these into the formula, the minimum sample size *n* = 3.8416∗0.5∗0.50.0025 = 384.16; a nonresponse of 5% = 5% × 384.16 = 19.21 was included. Therefore, the total number of caregivers who were sampled for the study was 384.16 + 19.21 = 403.37.

Eventually, 403 mothers or caregivers with children below 60 months were selected to respond to the questionnaire.

### 2.4. Data Collection and Analysis

Three research assistants received a 2-day training in data collection, research ethics, and interpretation of the questionnaire in Ewe and Akan. The research assistants had a minimum of a bachelor's degree and at least more than a year of experience in collecting data using a questionnaire. During the training, the researchers agreed on the terms they could use to describe concepts such as CWC, weighing, and vaccination in the local languages. This allowed for uniformity in the way the questions were asked. A respondent could prefer to respond to the questionnaire in any of the local languages.

A close-ended questionnaire made up of five sections (A–E) was used. Section “A” covered the sociodemographic characteristics, Section “B” covered the caregivers' knowledge on CWC, Section “C” covered the experiences, Section “D” covered the factors influencing CWC attendance, and Section “E” covered the barriers to attending CWC.

Permission was obtained from the community chiefs and elders, the local government, and the unit committee and assembly members to conduct the study. The questionnaire was pretested among 15 caregivers from the Titrinu community, and these caregivers were excluded from the main study. The pretested community had the same demographic characteristics as the study community. The pretesting determined the validity and reliability of the instrument and eliminated ambiguity in the questions. The test–retest reliability was computed using SPSS version 25 and recorded a minimum alpha Cronbach score of 0.71. The duration for administering the questionnaires was about 10–15 min.

All responses were cross-checked, coded, and entered into SPSS (version 25) for analysis. The data were analyzed using descriptive statistics and cross-tabulation using the univariate chi-square test. The statistically significant variables from the univariate analysis were then modeled using binary logistic regression. The level of significance was set at 5% (*p* value ≤ 0.05).

### 2.5. Ethical Considerations

Ethical clearance was obtained from the Research Ethics Committee (REC) of the Institute of Health Research of the University of Health and Allied Sciences. The study protocol, data collection, and handling adhered to the guidelines stipulated by the institutional ethics review committee. Participants' information and consent form were adequately explained to the study respondents, and all respondents provided written and verbal consent before they were enrolled.

## 3. Results

### 3.1. Sociodemographic Characteristics

The caregivers aged 31 to 35 (26.6%) were the largest group of participants, and the least were those above 40 years (4.5%). More than 50% had at least secondary education, and 17% had no formal education. A majority (76%) were gainfully employed, married (63.5%), living in rented houses (52.1%), and multiparous (85.1%). Those with children between 12 and 24 months were the largest group (37.2%).  Table 1 shows the distribution of demographic characteristics.

### 3.2. Caregivers Knowledge About Services Provided at CWCs

To determine the level of knowledge about the required CWC attendance among the caregivers, they were asked to indicate whether they agreed to specific statements or not (Table 2). A majority (98.5%) indicated that CWC sessions were to be attended once a month. With regard to the importance of attending CWC, a majority (97.5%) indicated CWC is important for growth monitoring, overall health monitoring of a child (97.3%), disease prevention (96.8%), and vaccination (94%). The caregivers were asked about the appropriate dates for a child to commence and complete CWC. The results showed that the majority (93.8%) indicated 6 weeks to 60 months. Nine of 10 caregivers agreed about the importance of continuously attending CWC after a child completes immunization. The caregivers agreed that the reasons for CWC attendance included growth monitoring (97.5%) and better child health monitoring (97.3%).

On child immunization, 90.3% indicated that BCG and OPV are given at birth, 84.9% indicated that OPV1, Pneumo 1, Rota 1, and Pentavalent are given at 6 weeks, and 84.9% indicated that Vitamin “A” (100,000 units) is given at 6 months. The caregivers also mentioned that measles–rubella and Vitamin “A” are given at 18 months (82.1%) and 12 months (79.7%); OPV2, Pneumo 2, Rota 2, Pentavalent (DPT–HepB–Hib) are given at 10 weeks (70.2%); measles–rubella and yellow fever are given at 9 months (66.7%); and OPV3, Pneumo 3, IPV, and Pentavalent (DPT–HepB–Hib) are given at 14 weeks (59.1%). Assessing the reasons for the continuous use of CWC services, the caregivers indicated nurses' recommendations (37.2%) and complying with the government's policy (29.8%). Table 2 shows the caregiver's knowledge on CWCs.

### 3.3. Predictors of Caregivers' Knowledge of CWC

The majority of the caregivers (80.4%) had high knowledge about CWC activities. Age, education, occupation, marital status, and parity were significantly associated with the level of knowledge of CWC activities. Those within the age groups 26 to 30 (COR = 2.63, CI: 1.6–87, *p* value = 0.044) and 31 to 35 (COR = 2.98, 95% CI: 1.13–7.83, *p* value = 0.023) were more likely to have good knowledge than those below 20 years. Those without formal education (COR = 0.34, 95% CI: 0.14–0.82, *p* value = 0.014) were less likely to have good knowledge than those with tertiary education. Those who are self-employed (COR = 2.05, 95% CI: 1.18–3.56, *p* value = 0.010) and government employed (COR = 3.22, 95% CI: 1.42–7.34, *p* value = 0.004) are more likely to have good knowledge than the unemployed. Those who were single (COR = 0.38, 95% CI: 0.23–0.64, *p* value < 0.001) were less likely to have good knowledge than those married (Table 3).

### 3.4. Experiences of Mothers About CWC

Most of the caregivers responded that they were happy anytime they took their children to the CWC (96.3%). The factors that influenced continued CWC attendance were encouragement by nurses (94.3%), nurses being empathetic (93.8%), showing a positive attitude toward caregivers (91.8%), having less waiting time (90.8%), and having appropriate timing for CWC services (90.1%). Others were caregivers' perception that attending CWC was an ideal childcare process (91.6%), knowing good childcare practices (90.6%), believing CWC leads to adequate care for sick children (89.8%), a perception that CWC is a needful care practice for children (82.4%), and perception that CWC is a requirement stated in the child welfare card (82.1%). The other factors that influenced mothers' continuous attendance at CWC included caregivers feeling embarrassed for nonattendance (79.0%) and observing child weight to be nonconforming with records on the CWC card (71.0%). The experiences of caregivers that promote regular CWC attendance include receiving health educational talks (69.5%), receiving adequate information before registration (68.2%), having adequate preparation before weighing (66.7%), immunization (62%), receiving individualized counseling (57.1%), and physical examination (37%).  Table 4 shows the distribution of factors associated with experiences of CWC attendance.

The overall experience of caregivers was reported to be good (74%). A multivariable logistic regression analysis showed a significant model. In this model, caregivers without formal education (AOR = 0.12, 95% CI: 0.03–0.41, *p* value = 0.001), primary education (AOR = 0.16, 95% CI: 0.05–0.50, *p* value = 0.002), or secondary education (AOR = 0.28, 95% CI: 0.09–0.80, *p* value = 0.021) were less likely to have good CWC experience compared with those who had tertiary level of education. Those who were self-employed were more likely to have good CWC experience compared with those employed by the government (AOR = 2.55, 95% CI: 1.02–6.40, *p* value = 0.044). Also, caregivers with good knowledge are more likely to have good CWC experience compared to those with poor knowledge (AOR = 3.51, 95% CI: 2.01–6.16, *p* value < 0.001). The factors that predicted good experiences of CWC are shown in Table 5.

### 3.5. Predictors of Regular CWC Attendance

The results indicated that 79.0% of the caregivers attended CWC sessions regularly. In the bivariate analysis, age, education, child's age, knowledge of CWC, and CWC experience of caregivers were associated with CWC attendance. Adjusting for confounding, the multiple logistic regression identified a significant likelihood ratio test (*p* value < 0.001) implying a good model fit. The adjusted odds ratio showed that caregivers without formal education (AOR = 0.10, 95% CI: 0.02–0.37, *p* value = 0.001), primary education (AOR = 0.13, 95% CI: 0.04–0.37, *p* value < 0.001), and secondary education (AOR = 0.34, 95% CI: 0.12–0.91, *p* value = 0.036) were less likely to have regular CWC attendance than those with tertiary education. Caregivers who were not employed were more likely to attend CWC sessions regularly compared with mothers who were employed. Experience at CWC (AOR = 2.52, 95% CI: 1.20–5.81, *p* value = 0.021) and having children between 0 and 11 months (AOR = 3.16, 95% CI: 1.50–6.89, *p* value = 0.003) were more likely to influence positively CWC attendance. The factors that predict regular CWC attendance are shown in Table 6.

## 4. Discussion

This study assessed the factors that influence CWC attendance among residents in a periurban community in the Volta Region of Ghana. CWC is an important health intervention that ensures immunization, comprehensive health education, growth monitoring, management of minor ailments, and vitamin supplementation. The result showed that caregivers with children less than 5 years old had good knowledge of CWC and the appropriate time to commence service. Having good knowledge is critical to people's likely adoption of a behavior that promotes positive health. However, in Ghana, knowledge of CWC attendance is relatively high within periurban communities, and attendance has been low [15]. Therefore, assessing the factors that are responsible for CWC attendance may be critical for identifying and implementing interventions to promote continued attendance. This is because knowledge of CWC must immediately translate to service acquisition and continued use. As a result, it was shown that the level of knowledge of caregivers influences their decisions to seek services [20]. In this study, we showed that the level of knowledge of caregivers regarding the activities at CWC has improved from that which was reported in other parts of Ghana [20, 21]. This improvement in the level of knowledge regarding services provided at the CWC may be attributable to improvement in the education given to mothers during the antenatal clinic (ANC) and CWC. The focus ANC services adopted in Ghana require that midwives provide adequate and comprehensive information about pregnancy, delivery, postnatal care, and CWC services [22].

The results showed that caregivers (80.4%) had a high knowledge of the activities at the CWC. Having a higher knowledge of CWC and related activities is critical for the mother's decision to use services [20–22]. However, with higher knowledge of services, use is still low. A previous study demonstrated lower knowledge with lower use of services [21]. It is therefore incumbent on service providers to identify the techniques for promoting the use of CWC to commensurate knowledge. Caregivers properly identified that CWC should continue until 60 months even after the completion of the routine immunization schedule. In the current study, the majority of mothers (93.8%) were aware that children should attend CWC until they are 5 years old. However, over a decade ago, in the Assin North community, mothers (95%) did not know how long a child should attend CWC after the completion of a routine immunization schedule [16]. These discrepancies in caregivers' knowledge within the last decade are indicative of the improvement made within Ghana's healthcare system regarding education on the appropriate time to complete CWC for children [22, 23]. The higher quality of education given to caregivers during CWC sessions may be responsible for the higher level of knowledge in the current study [23]. There is a need for healthcare providers and stakeholders to institute measures to promote collaboration among CWC planners to ensure that health education messages are tailored to benefit different cohorts of caregivers. It is imperative that in the current study, diverse factors appear to influence the likely use of CWC by caregivers. The majority of the caregivers identified appropriately that CWC was important and that regular attendance was critical for growth monitoring (97.5%), child's health (97.3%), and prevention of diseases (96.8%). The role and influence of CWC in the growth and development of children are critical for caregivers to have positive and acceptable expectations. Increasing knowledge of the details of services provided at CWC is critical for continued and sustained attendance [24]. Similarly, in Dansoman, Accra [25], and northern Ghana [20], most caregivers were knowledgeable of the potential benefits of CWC and the importance of CWC attendance. Caregivers must put in measures to ensure continued monitoring of children even after the completion of the immunization schedule [17]. The study further showed that those with a higher level of education (a minimum of tertiary level) had good knowledge with a more consistent attendance of CWC compared with their counterparts. This highlights the importance of educational levels on the health-seeking behavior of periurban communities. It is therefore imperative that healthcare interventions aimed at improving childcare target caregivers with relatively low levels of education.

We identified positive staff (nurses) attitude (93.8%) and high levels of satisfaction (91.8%) among caregivers to be responsible for the continuous attendance at CWC even after the completion of scheduled immunization. These findings depart from previous ones that identified negative staff attitudes as a critical hindrance to CWC attendance [16, 18]. The key reasons assigned to this negative attitude were that nurses were impatient to listen, publicly shamed women, lacked rapport with women, demonstrated a lack of professionalism, and had an insufficient sense of confidentiality, particularly during counseling sessions [18]. Caregivers promoted peer-to-peer education and encouraged supportive CWC attendance. In the current study, caregivers reported that nurses promoted regular attendance by minimizing the wait time at the clinic. This was an important measure as it was identified as a critical barrier to CWC attendance in the Ga Municipality [18]. Also, marital status and CWC knowledge were critical factors that promoted the use of CWC. In previous studies, one important factor that influenced the use of CWC was marital status [15, 16]. This may be attributable to the relative stability of married couples psychologically and the possible encouragement gotten from husbands and/or family members.

We identified multifaceted reasons responsible for the nonattendance of CWC by caregivers. This current study showed diverse sociodemographic, client-centered, service-provider-related, and systematic factors that influence knowledge, experience, and CWC attendance among caregivers in a periurban community. The barriers to noncontinuous attendance of CWC were the busy schedules of caregivers, children starting school, the perception that the child is old enough to quit CWC, caregivers' forgetfulness, traveling outside the town, caregivers' laziness to go to CWC, language barrier, long distance to service centers, and attitude of the healthcare providers. These factors may be grouped as caregiver-related factors, child-related factors, healthcare-related factors, and systematic-related factors. Measures must be initiated to address the barriers to CWC attendance by segregating and addressing specific barriers based on proper classification. To promote CWC attendance in periurban areas, researchers must tailor healthcare interventions to mitigate these barriers [16, 26, 27]. Previous studies also identified these reasons as barriers to CWC attendance in rural, urban, and periurban areas [15, 16, 26]. Previous studies identified busy schedules [26], completion of immunization schedules [16], children starting school [16], children old enough to stop [16, 27], long distance [16, 28, 29], poor staff/nurse attitude [15, 16], and longer or protracted wait time [30] as the barriers to CWC attendance. It is therefore imperative that given the importance of CWC in a child's development, measures are immediately instituted to promote attendance even after the completion of the routine immunization schedule.

### 4.1. Strengths and Limitations

This study identified demographic factors that influence knowledge, experience, and CWC attendance among caregivers in a periurban community in the Volta Region. One important strength is that this was one of the very first community-based studies in the Godokpe community to assess the factors associated with CWC attendance among caregivers. Also, to the best of our knowledge, this study is one of the first targeting caregivers of children under 5 in the current settings and will serve as a basis for implementing interventions to ensure continuous use of CWC services even when the child has completed all the immunization schedules. However, this study is not without some limitations. One critical limitation of this study is that it did not account for caregivers' beliefs and cultural inclinations as determining factors for CWC attendance, knowledge, or use. Also, the questionnaire did not identify the reasons for the nonattendance of CWC; hence, this was only extrapolated. Future studies in this area may need to consider these factors in design. Another important limitation of this study was the fact that the data collection was collected in English, Ewe, and Akan, introducing the likelihood of variability in the interpretation of the questionnaire by different respondents (a possible bias). However, this was mitigated by the researchers because, before data collection, they agreed on the specific words to use to describe key terms in the various local languages. Future research methods can consider creating codes/terms in local languages in Ghana that can be used among researchers.

## 5. Conclusion

We identified multiple and interrelated factors that influence the level of knowledge, nature of experience, and attendance of CWC in a periurban community in the Volta Region of Ghana. As the knowledge level was consistently high, caregivers did not continue attendance at CWC. This justifies the use of diverse behavior change techniques by researchers to promote consistent use after the completion of the immunization schedule. Also, nursing administrators must make and implement policies encouraging community health nurses to conduct home visiting services for CWC service provision, especially for those who have completed the recommended scheduled vaccination until they are up to 60 months. Also, future research must segregate caregivers and nature and barriers to CWC. This will help them to identify efficient means of promoting behavior change and encouraging continued CWC attendance even after the completion of routine childhood [31] immunizations.

## Figures and Tables

**Table 1:** Distribution of demographic characteristics.

Variable	Response	Frequency	Percentage
Age	≤ 20	25	6.2
	21 to 25	102	25.3
	26 to 30	102	25.3
	31 to 35	107	26.6
	36 to 40	49	12.2
	41+	18	4.5

Education	No formal education	63	15.6
	Primary	154	38.2
	Secondary	120	29.8
	Tertiary	66	16.4

Occupation	Self-employed (private sector)	235	58.3
	Government employed (public sector)	74	18.4
	Unemployed	94	23.3

Marital status	Widowed/separated/divorced	17	4.2
	Single	130	32.3
	Married	256	63.5

Residence	Rented	210	52.1
	Self-owned	193	47.9

Parity	Nulliparous/primiparous	5	1.2
	Low multiparous (1 to 3)	343	85.1
	Grand multiparous (4 to 8)	55	13.6

Child's age	0–11 months	117	29.0
	12–24 months	150	37.2
	24–59 months	136	33.8

**Table 2:** Caregiver's knowledge of child welfare clinic.

Variables	Responses	Yes	No
*Knowledge on CWC*			
Frequency of CWC	CWC sessions are to be attended once a month	397 (98.5)	6 (1.5)
	Begins at 6 weeks and ends at 59 months	378 (93.8)	25 (6.2)
	Important even after immunization completion	373 (92.6)	30 (7.4)
Importance of regular attendance CWC	Growth monitoring	393 (97.5)	10 (2.5)
	Monitoring child health	392 (97.3)	11 (2.7)
	Prevention of diseases	390 (96.8)	13 (3.2)
	Weighed before vaccination	379 (94)	24 (6)
	Caregivers care for the child	369 (91.6)	34 (8.4)

*Knowledge of immunization at CWC*			
Birth	BCG and OPV are given at birth	364 (90.3)	39 (9.7)
6 weeks	OPV1, Pneumo 1, Rota 1, and Pentavalent	342 (84.9)	61 (15.1)
10 weeks	OPV2, Pneumo 2, Rota 2, Pentavalent	283 (70.2)	120 (29.8)
14 weeks	OPV3, Pneumo 3, IPV, and Pentavalent	238 (59.1)	165 (40.9)
6 months	Vitamin A (100,000 units) is given at 6 months	342 (84.9)	61 (15.1)
9 months	Measles–rubella and yellow fever	269 (66.7)	134 (33.3)
12 months	Vitamin A (200,000 units)	321 (79.7)	82 (20.3)
18 months	Measles–rubella and vitamin A	331 (82.1)	72 (17.9)
Reasons for compliance	Nurses' recommendation	150 (37.2)	253 (62.8)
	Complying with the government's policy	120 (29.8)	283 (70.2)

**Table 3:** Correlates of the knowledge level of participants.

Variables	Yes	No	*p* value	COR (95% CI)
Age				
≤ 20	16	9		
21 to 25	78	24	0.203	1.83 (0.72, 4.66)
26 to 30	84	18	0.044	2.63 (1, 6.87)
31 to 35	90	17	0.023	2.98 (1.13, 7.83)
36 to 40	41	8	0.057	2.88 (0.95, 8.78)
41+	14	4	0.332	1.97 (0.5, 7.82)
Educational level				
No formal education	43	20	0.014	0.34 (0.14, 0.82)
Primary	119	35	0.122	0.54 (0.24, 1.19)
Secondary	104	16	0.594	1.03 (0.43, 2.47)
Tertiary	57	9		
Occupation				
Self-employed	193	42	0.010	2.05 (1.18, 3.56)
Government employed	65	9	0.004	3.22 (1.42, 7.34)
Unemployed	65	29		
Marital status				
Widowed/separated/divorced	12	5	0.086	0.39 (0.13, 1.18)
Single	91	39	< 0.001	0.38 (0.23, 0.64)
Married	220	36		
Residence				
Rented	166	44	0.563	0.87 (0.53, 1.41)
Self-owned	157	36		
Parity				
Nulliparous/primiparous	1	4	0.021	0.10 (0.01, 0.99)
Low multiparous (1 to 3)	283	60	0.042	1.94 (1.02, 3.69)
Grand multiparous (4 to 8 or above)	39	16		
Child's age				
0–11 months	96	21	0.222	1.47 (0.79, 2.71)
12–24 months	124	26	0.148	1.53 (0.86, 2.72)
24–59 months	103	33		

**Table 4:** Distribution of factors associated with experiences of CWC attendance.

Experience level	Yes (%)	No (%)
Happy to attend CWC	388 (96.3)	15 (3.7)
Encouragement by nurses	380 (94.3)	23 (5.7)
Nurses were empathetic	378 (93.8)	25 (6.2)
The positive general attitude of nurses	370 (91.8)	33 (8.2)
Ideal way of childcare	369 (91.6)	34 (8.4)
Less waiting time	366 (90.8)	37 (9.2)
Knowledge of childcare practices	365 (90.6)	38 (9.4)
Appropriate timing of CWC	363 (90.1)	40 (9.9)
Care of sick children	362 (89.8)	41 (10.2)
The need for CWC services	332 (82.4)	71 (17.6)
Educated based on the information card	331 (82.1)	72 (17.9)
Embarrassed by nonattendance	83 (20.6)	320 (79.4)
Blame by nurses for nonattendance	116 (28.8)	287 (71.2)
Health educational talk	280 (69.5)	123 (30.5)
Adequate information before registration	275 (68.2)	128 (31.8)
Adequate preparation before weighing	269 (66.7)	134 (33.3)
Immunization	250 (62)	153 (38)
Counseling	230 (57.1)	173 (42.9)
Physical examination	149 (37)	254 (63)

**Table 5:** Correlates of the experiences of CWC of mothers.

Variables	Yes	No	*p* value	COR (95% CI)	*p* value	AOR (95% CI)
Age						
≤ 20	17	8				
21 to 25	72	30	0.800	1.13 (0.44, 2.90)	0.817	0.88 (0.29, 2.56)
26 to 30	77	25	0.444	1.45 (0.56, 3.76)	0.979	1.02 (0.31, 3.15)
31 to 35	82	25	0.369	1.54 (0.60, 4.00)	0.799	0.86 (0.25, 2.80)
36 to 40	38	11	0.374	1.63 (0.56, 4.77)	0.772	1.21 (0.33, 4.42)
41+	11	7	0.640	0.74 (0.21, 2.62)	0.724	0.75 (0.15, 3.69)
Education						
No education	38	25	0.001	0.24 (0.10, 0.57)	0.001	0.12 (0.03, 0.41)
Primary	108	46	0.011	0.37 (0.17, 0.81)	0.002	0.16 (0.05, 0.50)
Secondary	94	26	0.180	0.57 (0.25, 1.30)	0.021	0.28 (0.09, 0.80)
Tertiary	57	9				
Occupation						
Self-employed	175	60	0.657	0.87 (0.47, 1.61)	0.044	2.55 (1.02, 6.40)
Government employed	57	17				
Unemployed	65	29	0.256	0.67 (0.33, 1.34)	0.055	2.83 (0.98, 8.29)
Marital status						
Widowed/separated/divorced	10	7	0.083	0.42 (0.15, 1.15)		
Single	89	41	0.059	0.64 (0.40, 1.02)		
Married	198	58				
Residence						
Rented	158	52	0.464	1.18 (0.76, 1.84)	0.37	1.25 (0.77, 2.06)
Self-owned	139	54				
Parity						
Nulliparous/primiparous	1	4	0.021	0.10 (0.01, 0.99)	0.067	0.11 (0.01, 0.91)
Low multiparous	257	86	0.526	1.23 (0.65, 2.30)	0.374	0.71 (0.33, 1.47)
Grand multiparous	39	16				
Child's age						
0–11 months	86	31	0.254	1.37 (0.80, 2.36)	0.45	1.28 (0.67, 2.46)
12–24 months	120	30	0.012	1.98 (1.16, 3.38)	0.076	1.74 (0.95, 3.23)
24–59 months	91	45				
Overall knowledge						
Good	257	66	< 0.001	3.89 (2.33, 6.52)	< 0.001	3.51 (2.01, 6.16)
Poor	40	40				

**Table 6:** Correlates of CWC attendance.

Variables	Yes	No	*p* value	COR (95% CI)	*p* value	AOR (95% CI)
Age						
≤ 20	10	15				
21 to 25	21	81	0.043	0.39 (0.15, 0.99)	0.100	0.40 (0.13, 1.21)
26 to 30	20	82	0.031	0.37 (0.14, 0.93)	0.152	0.42 (0.13, 1.38)
31 to 35	20	87	0.022	0.35 (0.14, 0.88)	0.053	0.29 (0.08, 1.02)
36 to 40	11	38	0.113	0.43 (0.15, 1.23)	0.433	0.59 (0.16, 2.20)
41+	2	16	0.037	0.19 (0.04, 1.00)	0.519	0.52 (0.06, 3.44)
Education						
No formal education	9	54	0.011	0.33 (0.14, 0.80)	0.001	0.10 (0.02, 0.37)
Primary	22	132	0.001	0.33 (0.17, 0.66)	< 0.001	0.13 (0.04, 0.37)
Secondary	31	89	0.278	0.70 (0.36, 1.34)	0.036	0.34 (0.12, 0.91)
Tertiary	22	44				
Occupation						
Government employed	17	57				
Self-employed	42	193	0.33	0.73 (0.39, 1.38)	0.074	2.48 (0.95, 7.10)
Unemployed	25	69	0.590	1.22 (0.60, 2.47)	0.014	4.35 (1.39, 14.61)
Residence						
Rented	39	171	0.241	0.75 (0.46, 1.22)	0.503	0.83 (0.49, 1.42)
Self-owned	45	148				
Parity						
Grand multiparous	6	49				
Low multiparous	77	266	0.051	2.36 (0.98, 5.73)	0.530	1.37 (0.55, 3.97)
Primiparous	1	4	1.00	2.04 (0.20, 21.40)	0.249	4.69 (0.18, 52.24)
Child's age						
0–11 months	36	81	< 0.001	3.59 (1.84, 6.97)	0.003	3.16 (1.50, 6.89)
12–24 months	33	117	0.013	2.28 (1.18, 4.41)	0.092	1.85 (0.92, 3.88)
24–59 months	15	121				
Knowledge						
Yes	74	249	0.040	2.08 (1.02, 4.24)	0.163	1.80 (0.82, 4.35)
No	10	70				
Experience						
Yes	74	223	0.001	3.19 (1.58, 6.43)	0.021	2.52 (1.20, 5.81)
No	10	96				

## Data Availability

The data that support the findings of this study are available on request from the corresponding author. The data are not publicly available due to privacy or ethical restrictions.
